# Simple and economical biosensors for distinguishing *Agrobacterium*-mediated plant galls from nematode-mediated root knots

**DOI:** 10.1038/s41598-019-54568-2

**Published:** 2019-11-29

**Authors:** Okhee Choi, Juyoung Bae, Byeongsam Kang, Yeyeong Lee, Seunghoe Kim, Clay Fuqua, Jinwoo Kim

**Affiliations:** 1 Institute of Agriculture & Life Science, Gyeongsang National University, Jinju, 52828 Republic of Korea; 2 Department of Plant Medicine, Gyeongsang National University, Jinju, 52828 Republic of Korea; 3 Division of Applied Life Science, Gyeongsang National University, Jinju, 52828 Republic of Korea; 4 Department of Biology, Indiana University, Bloomington, IN 47405 USA

**Keywords:** Bacterial genes, Bacterial genetics

## Abstract

*Agrobacterium*-mediated plant galls are often misdiagnosed as nematode-mediated knots, even by experts, because the gall symptoms in both conditions are very similar. In the present study, we developed biosensor strains based on agrobacterial opine metabolism that easily and simply diagnoses *Agrobacterium*-induced root galls. Our biosensor consists of *Agrobacterium* mannitol (ABM) agar medium, X-gal, and a biosensor. The working principle of the biosensor is that exogenous nopaline produced by plant root galls binds to NocR, resulting in NocR/nopaline complexes that bind to the promoter of the nopaline oxidase gene (*nox*) operon and activate the transcription of *noxB*-*lacZY*, resulting in readily visualized blue pigmentation on ABM agar medium supplemented with X-gal (ABMX-gal). Similarly, exogenous octopine binds to OccR, resulting in OoxR/octopine complexes that bind to the promoter of the octopine oxidase gene (*oox*) operon and activate the transcription of *ooxB*-*lacZY*, resulting in blue pigmentation in the presence of X-gal. Our biosensor is successfully senses opines produced by *Agrobacterium*-infected plant galls, and can be applied to easily distinguish *Agrobacterium* crown gall disease from nematode disease.

## Introduction

Plant root galls are abnormal root tissue outgrowths that are caused by various parasites, including viruses, fungi, microscopic soil nematodes, and bacteria. Economic losses due to damage by nematodes and bacteria are significant; however, their galls are difficult to distinguish. Root-knot nematodes (*Meloidogyne* spp.) cause approximately 5% of global crop losses in over 2,000 susceptible plants^1^. Crown galls caused by *Agrobacterium tumefaciens* occur worldwide, causing great damage annually. This rod-shaped Gram-negative soil bacterium belongs to the class α-proteobacterium, and causes galls on the roots of susceptible plants^2^. The *A*. *tumefaciens* host range is often very broad; the bacterium causes crown galls that compromise the commercialization of plants in more than 60 families, including dicotylodonous plants, ornamental plants, brambles, and stone fruit and pome trees^3–6^.

The accurate and rapid diagnosis of crown gall is difficult but important for control and quarantine. There have been various attempts to diagnose and detect *A*. *tumefaciens* from plant tumors, including isolation using selective media, pathogenicity tests, biochemical and physiological tests, serological assays using antibodies, and polymerase chain reaction (PCR) techniques. In previous studies, the use of selective media for different biovars has allowed successful *A*. *tumefaciens* isolation^7–9^. However, this method of *A*. *tumefaciens* isolation is difficult and time-consuming. Several studies have applied serological techniques for *A*. *tumefaciens* detection, but this method has not been useful for the detection of pathogenic strains^10,11^. PCR techniques are the most frequently used methods for *A*. *tumefaciens* detection and diagnosis. Methods to diagnose crown galls with PCR have been developed in various ways^12–14^. *Rhizobium* and *Agrobacterium* are very similar in many respects, and it is difficult to distinguish these genera using PCR-based assays. Some studies have found no difference between *Rhizobium* and *Agrobacterium* in phylogenetic studies using 16S rRNA gene sequence. One method used to differentiate *Agrobacterium* from *Rhizobium* is to determine whether the bacteria induce pathogenic symptoms or root nodules; these symptoms are plasmid dependent. Thus, much effort has been made to avoid confusion between *Rhizobium* and *Agrobacterium* by designing the primer pairs used in PCR based on the gene located on the Ti (tumor-inducing) plasmid of *A*. *tumefaciens*. Another fundamental problem of the PCR method is that bacterial pathogens generally multiply into large populations in diseased lesions, but crown gall bacterial pathogens persist at significantly lower populations away from lesions and PCR-mediated diagnosis in plant samples requires a threshold population density. A major limitation of routine PCR application to diagnose plant disease is that successful PCR can be prevented by the frequent occurrence of polyphenolic inhibitors and thermostable DNA polymerase inhibitors in plant tissues^12,15–17^. To overcome these problems, a new approach to crown gall diagnosis is needed. Specifically, there is an urgent need for the development of new, simple, and rapid molecular-based diagnostic techniques. Therefore, in this study, we developed an easy and simple *Agrobacterium* biosensor based on opine catabolism.

To diagnose *Agrobacterium* crown galls, it is important to understand their complex opine biology. *A*. *tumefaciens* pathogenicity is initiated by transferring a segment comprising roughly 20% of the Ti plasmid, called the T-DNA (~40 kb) into plant cells during infection^3,18^. Genes in the transferred DNA are expressed in the plant nucleus, and are responsible for inducing tumorous growth of the transformed cells and for synthesizing opines, which serve as nutrient for *A*. *tumefaciens* that colonize the infected tissue^19^. Two very common opines are octopine and nopaline, which are produced in plant cells transformed with *A*. *tumefaciens* which harbor octopine- and nopaline-type Ti plasmids, respectively^20^. Opine biosynthetic genes on the T-DNA are distinct from their catabolic genes. Opine produced by transformed plant cells stimulate the expression of catabolic genes that are carried in the non-transferred portion of the *A*. *tumefaciens* Ti plasmid^21^. Nopaline tumors are caused by T-DNA transfer from nopaline-utilizing strains, and octopine tumors are caused by T-DNA transfer from strains that metabolize octopine^22^. In contrast, one group of strains can utilize both nopaline and octopine, although their tumors synthesize only nopaline, and another group utilizes nopaline, but their tumors produce either nopaline or octopine^23^. Additionally, some strains can utilize both types of opines, but their tumors produce neither nopaline nor octopine^21–24^. The *nox* or *oox* regions of the pTiC58 (nopaline-type) or pTi15955 (octopine-type) Ti plasmids are responsible for the catabolic utilization of nopaline or octopine in the *A*. *tumefaciens* strains C58 or 15955, respectively^20,24^. Catabolic functions are activated in the presence of exogenous nopaline or octopine, and regulatory controls are mediated by the LysR-type transcriptional regulatory proteins NocR or OccR; the genes encoding these proteins are located in the opine transporter regions (*noc* and *occ*) of the C58 and 15955 strains, respectively^24,25^.

The aims of the present study were to (1) develop a method to diagnose crown gall using opine detection and (2) evaluate the sensitivity of this method in practical application. Conventional density-based diagnostic methods for other bacterial plant diseases are not suitable for crown gall diagnosis. Therefore, a new diagnostic method was developed using opine metabolic genes on the Ti plasmid of *Agrobacterium*. Our diagnosis method is based on sensing the presence of external opines, and allows visualization of the expression of opine catabolism genes. The engineered opine-responsive, regulator based bacterial biosensors will be helpful for researchers and plant growers to detect plant diseases caused by *A*. *tumefaciens*, and will reduce detection time and cost. These biosensors can also be applied to evaluate and quantify opines produced by plant gall tissues.

## Results and Discussion

**Figure 1:**
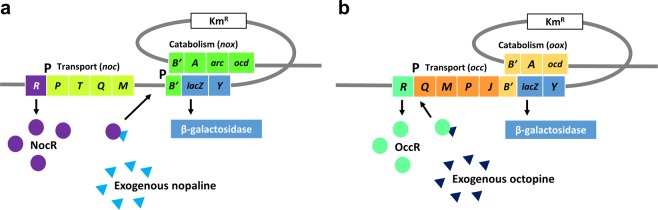
Working model of the opine-based biosensor strains. (**a**) Working model of the nopaline-based biosensor strain. Exogenous nopaline binds to NocR, a LysR-type transcriptional activator; the NocR/nopaline complex activates *noxB*-*lacZY* transcription, resulting in β-galactosidase expression. (**b**) Working model of the octopine-based biosensor strain. Exogenous octopine binds to OccR, a LysR-type transcriptional activator; the OccR/octopine complex activates *ooxB*-*lacZY* transcription, resulting in β-galactosidase expression.

Two plant samples showing root gall symptoms were delivered to our laboratory by farmers in 2018, one from squash and the other from raspberry. The farmers questioned whether their crops were infested with nematodes or bacteria. Nematode-mediated knots are often mistaken for *Agrobactgerium*-mediated galls, because the symptoms of both infections are difficult to distinguish, even for experts. General identification and detection of plant pathogens from plant materials typically requires 1–2 weeks, followed by an additional 3–4 weeks of pathogenicity tests. An easy and fast detection method is needed for local extension services. Although current PCR techniques have opened a new era of pathogen diagnosis, their application and interpretation can be complex. PCR techniques require PCR machinery, polymerases, buffers, and oligonucleotide primers; visualization of the results can also require UV lamps, ethidium bromide, and electrophoresis. Thus, we developed a simple molecular *Agrobacterium* biosensor detection method, based on two engineered, opine-responsive *A*. *tumefaciens* derivatives. As shown in Fig. 1, exogenous nopaline binds to NocR, a LysR-type transcriptional activator. The resulting NocR/nopaline complex activates the transcription of *noxB*-*lacZY*, resulting in β-galactosidase expression (Fig. 1a). Similarly, exogenous octopine binds to OccR, a LysR-type transcriptional activator, and the resulting OccR/octopine complex activates *ooxB*-*lacZY* transcription, resulting in β-galactosidase expression (Fig. 1b)^23,25^.

### Construction of opine biosensor strains

Nopaline and octopine catabolism operons carry genes responsible for transport and catabolism. To allow the entry of external opines, genes responsible for opine transport must remain intact and be active. However, disruption of the first cytoplasmic step of opine catabolism does not prevent transport of the opine into the cell, and opine-responsive gene regulation would be maintained. Thus, opine catabolism genes encoding opine oxidase were targeted for *lacZY* reporter fusions, and *noxB* of *A*. *tumefaciens* C58 and *ooxB* of *A*. *tumefaciens* 15955 simultaneously disrupted and fused to the *lacZY* reporter via Campbell integration as described previously^26^. The internal fragment of the target gene was inserted into pVIK112. The plasmids were then transferred from S17-1/λ*pir* into the *A*. *tumefaciens* strain C58 or 15955 by conjugation, selecting for kanamycin-resistant target–*lacZY* transcriptional fusion. The expression levels of *noxB*-*lacZY* and *ooxB*-*lacZY* were visualized using X-gal or ONPG when nopaline and octopine were provided exogenously.

### Responses of opine biosensor strains to synthetic opines

**Figure 2:**
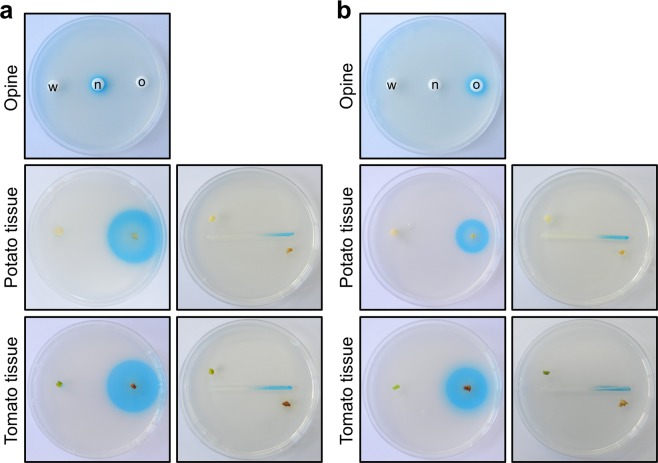
Responses of the opine biosensor strains to synthetic opines, potato and tomato tumor tissues induced by the *A*. *tumefaciens* strain C58 or 15955. (**a**) Responses of the nopaline-based biosensor (C58 *noxB*-*lacZY*) to synthetic opines, potato and tomato tumor tissues induced by the *A*. *tumefaciens* strain C58. (**b**) Responses of the octopine-based biosensor (15955 *ooxB*-*lacZY*) to synthetic opines, potato and tomato tumor tissues induced by the *A*. *tumefaciens* strain 15955. Blue color around paper discs containing opines (10 nM) and a biosensor strain embedded in ABMX-gal plates indicates the presence of opines and β-galactosidase expression. w, Water control; n, nopaline; and o, octopine. Blue color around plant tumor tissues (right side on each plate) on a biosensor embedded in ABMX-gal indicates the presence of opines and β-galactosidase expression. Healthy plant tissues exhibited no color (left side on each plate). The biosensor functioned well as an opine sensor.

Synthetic opines were used to determine whether the opine biosensor strains were functional as predicted. A blue ring was observed around a paper disc containing nopaline and the C58 *noxB*-*lacZY* biosensor strain embedded in ABMX-gal, indicating the presence of nopaline and β-galactosidase expression (Fig. 2a). Similarly, a blue ring was observed around a paper disc containing octopine and the 15955 *ooxB*-*lacZY* biosensor strain embedded in ABMX-gal, indicating the presence of octopine and β-galactosidase expression (Fig. 2b). Neither biosensor responded detectably to the non-cognate opine (octopine for *noxB*-*lacZY* and nopaline for *ooxB*-*lacZY*).

### Responses of opine biosensor strains to plant tumor tissues

To determine whether the opine-based biosensors responded to plant opines, tumor tissue was induced in potato and tomato and used for plant assays. Plant tumor tissue samples induced with *A*. *tumefaciens* strains C58 or 15955 and healthy plant tissues (negative control) were placed on biosensor-imbedded ABMX-gal or on ABMX-gal agar plates close to streaks of the biosensor strain. The nopaline-based biosensor (C58 *noxB*-*lacZY*) exhibited a blue color in response to plant tumor tissues induced by *A*. *tumefaciens* C58 (Fig. 2a). The octopine-based biosensor (15955 *ooxB*-*lacZY*) exhibited a blue color in response to plant tumor tissues induced by *A*. *tumefaciens* 15955 (Fig. 2b). Again, each biosensor was specific; the *nonB*-*lacZY* biosensor only responded to tissue infected by the nopaline strain C58, not tissue infected with 15955, and the *ooxB*-*lacZY* only responded to the tissue infected with 15955. The uninfected plant tissue controls also resulted in no color changes.

### Thin layer chromatography assay

**Figure 3:**
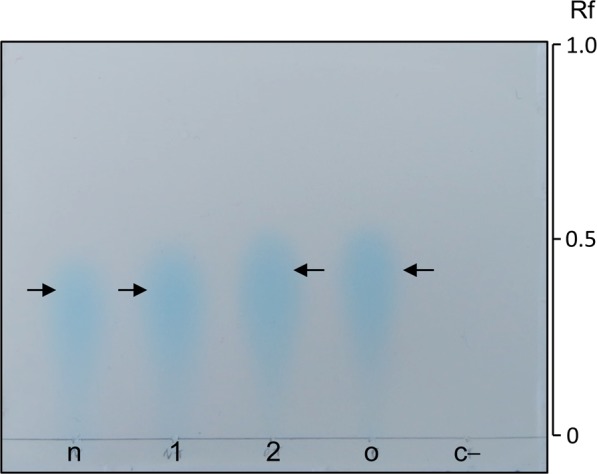
Thin-layer chromatography (TLC) analysis of opines using the biosensor strains. Reverse-phase TLC plates (12 × 10 cm) were developed using chloroform:acetic acid:water (2.8:3.5:0.7, v:v:v) and overlaid with ABMX-gal containing the nopaline- and octopine-based biosensor strains (C58 *noxB*-*lacZY* and 15955 *ooxB*-*lacZY*, respectively). n, Synthetic nopaline; 1, extract of tomato tumor induced by C58; 2, extract of tomato tumor induced by 15955; o, synthetic octopine; and c–, extract of healthy tomato tissue (negative control). Rf, retardation factor.

To determine the type of opines present with *Agrobacterium* strains, plant opines were historically subjected to paper electrophoresis; the devices used in this type of electrophoresis are not currently used or readily available. We therefore attempted to perform opine analysis using TLC plates, which were readily obtained. Crude extracts from tomato tumor tissue samples or synthetic nopaline and octopine were loaded onto a reverse-phase TLC system. Once developed these plates were dried and overlaid with a suspension containing both the nopaline and the octopine biosensor strains in ABMX-gal agar. After incubation, inverted treardrop-shaped regions of light blue pigmentation were observed for all of the opine-containing samples. The retardation factor (Rf) of nopaline (0.35) and octopine (0.4) were comparable. We also confirmed nopaline and octopine could be detected in extracts from infected plants, and appeared identical to the synthetic opines by TLC (Fig. 3). This study is the first to perform TLC-based opine analysis, which will be helpful to researchers performing similar studies.

### Quantification of opines based on β-galactosidase activity assay

**Figure 4:**
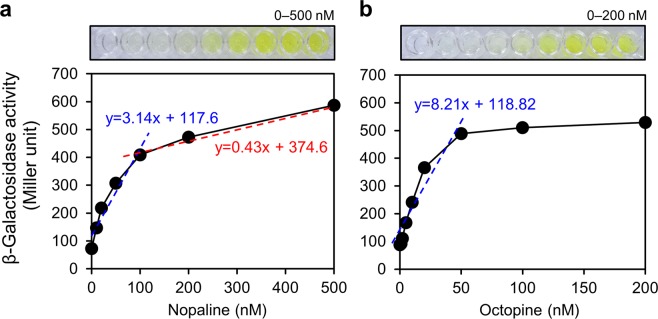
Plots of the influence coefficient of β-galactosidase activity *versus* the opine concentration of the proposed biosensors. β-galactosidase activity responses to (**a**) nopaline and (**b**) octopine. The results of the nopaline-based biosensor (C58 *noxB*-*lacZY*) exhibited a good linear relationship in the ranges of 0–100 (blue) and 100–500 nM (red) with different slops. The octopine-based biosensor (15955 *ooxB*-*lacZY*) showed even better sensitivity for octopine detection than the nopaline biosensor for nopaline, with a linear relationship in the ranges of 0–50 nM (blue). Error bars represent the standard deviations of three independent measurements. The distinct yellow color of *o*-nitrophenol from the ONPG substrate in microplates indicates the presence of opines.

We then evaluated the sensitivity of opine-based biosensors by measuring β-galactosidase enzyme activity using ONPG as a substrate. The linear dose response was calculated using synthetic nopaline and octopine. The results of the nopaline-based biosensor (C58 *noxB*-*lacZY*) exhibited a good linear relationship in the ranges of 0–100 and 100–500 nM, with a greater slope for the lower concentration range (0–100 nM) than for the higher concentration range (100–500 nM) (Fig. 4a). The octopine-based biosensor (15955 *ooxB*-*lacZY*) showed even better sensitivity for octopine detection (Fig. 4b) than the nopaline biosensor for nopaline, with a linear relationship in the ranges of 0–50 and 50–200 nM, with slopes following the same pattern exhibited by C58 *noxB*-*lacZY*. The octopine-based biosensor showed excellent sensitivity to octopine detection, and higher sensitivity than the nopaline-based biosensor.

### Application of biosensor strains for gall diagnosis

**Figure 5:**
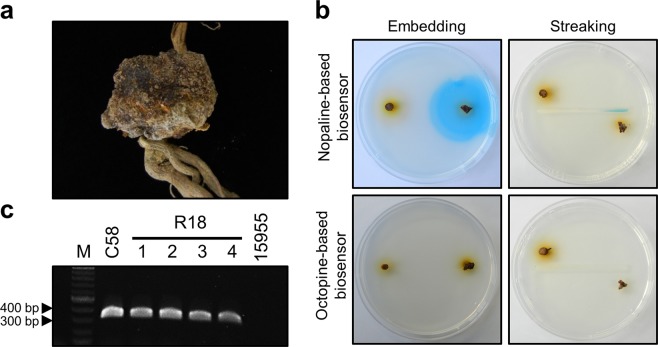
Responses of the biosensor strains to raspberry galls. (**a**) Photograph of natural raspberry root gall symptoms. (**b**) Plate-based biosensor assays. Blue color around raspberry gall tissues (right side of each plate) indicates the presence of nopaline and β-galactosidase expression. Healthy raspberry tissues resulted in no color change (left side of each plate) in the octopine-based biosensor. (**c**) Nopaline type T-DNA fragment amplification of isolate R18 (1–4 colonies) from raspberry gall using RBF and RBR primers. M, 100-bp ladder marker; C58, *A*. *tumefaciens* strain; 15955, *A*. *tumefaciens* strain. The raspberry root gall was diagnosed as being caused by nopaline-type *A*. *tumefaciens*.

**Figure 6:**
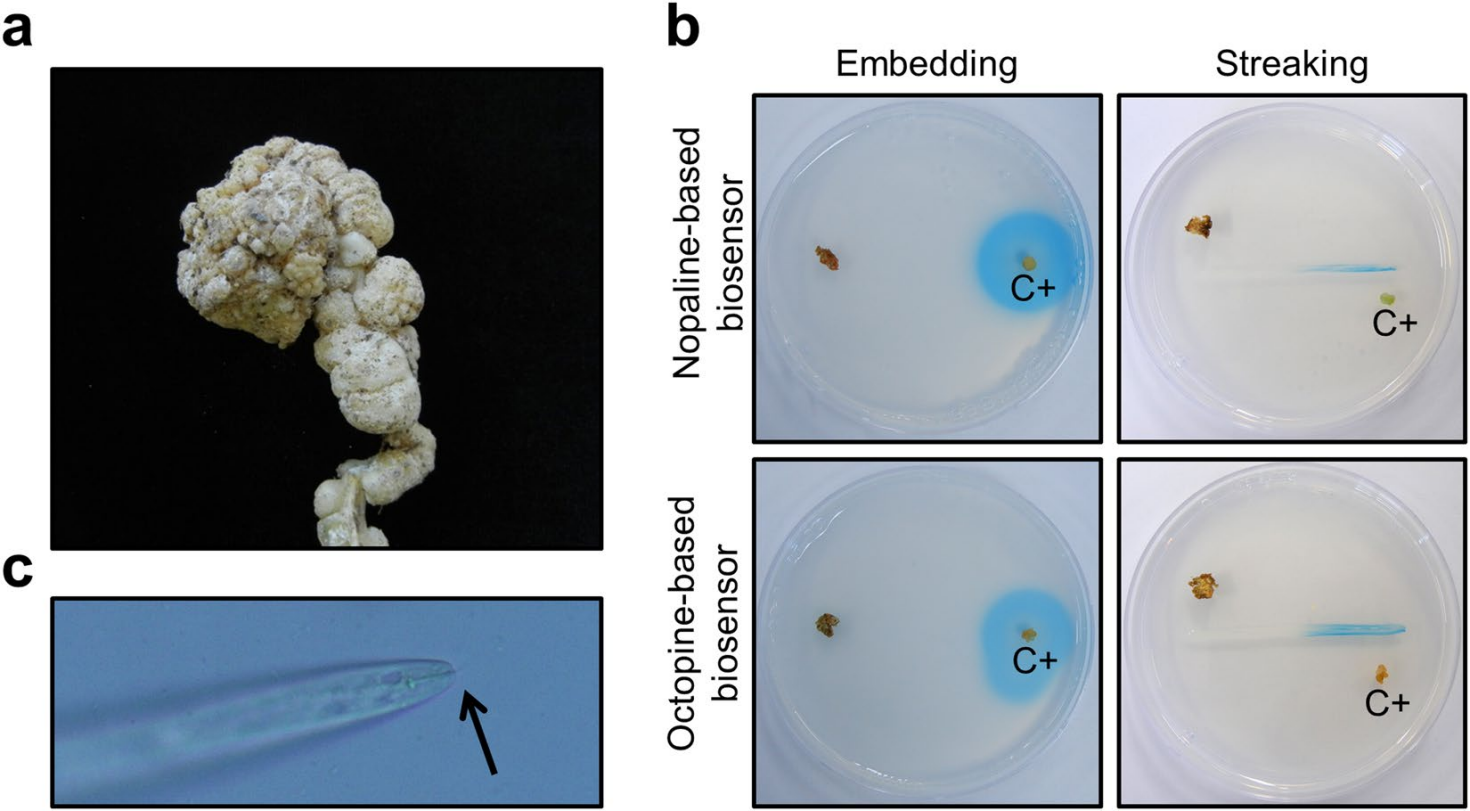
Responses of the biosensors to squash root galls. (**a**) Photograph of natural squash root gall symptoms. (**b**) No response around squash root gall tissues (left side of each plate) on both nopaline- and octopine-based biosensors indicates the absence of opines. Positive control potato disc tumors (C+) artificially induced by *Agrobacterium* strains C58 (upper) or 15955 (lower) exhibited blue color. (**c**) Micrographs of root-knot nematode isolated from the squash samples. The arrow indicates the stylet. The squash root gall was suspected to have been caused by root knot nematodes.

The gall symptoms of the raspberry and squash samples that prompted this study were very similar (Figs. 5a and 6a). Both samples were diagnosed using the biosensor strains developed in this study. The raspberry gall samples exhibited a blue color in the nopaline-based biosensor, but no reaction in the octopine-based biosensor (Fig. 5b). The raspberry gall was ultimately confirmed to be a crown gall and diagnosed as a nopaline-type *A*. *tumefaciens* infection. Although the diagnosis was completed using the biosensor developed in this study, we isolated the causative bacterium from the root gall tissues to perform a PCR test using the bacterial DNA and the RBF (5′-TGACAGGATATATTGGCGGGTAA-3′) and RBR (5′-TGCTCCGTCGTCAGGCTTTCCGA-3′) primer set designed based on the nopaline type T-DNA right border^14^. The *A*. *tumefaciens* strains from the raspberry crown gall, which included C58 and R18 (four colonies, 1–4), exhibited the anticipated bands. The octopine type strain 15955 resulted in no bands (Fig. 5c).

The squash gall samples were examined in a similar manner; they resulted in no color changes in either the nopaline- or octopine-based biosensor (Fig. 6b). Only positive controls caused blue color to form. Microscopic examination of the squash root gall tissues revealed root knot nematodes possessing a stylet (Fig. 6c).

A previous study compared the expression of the pathogenic *A*. *tumefaciens* gene *pinF* using enzyme activity assays at different concentrations of nopaline and octopine^21^. The opine concentrations and enzyme activity levels (Miller unit) were different in the present study from those reported previously^21^, likely due to differences in the expression levels of each gene. Millimolar-level amounts (mM) of opines are required for *pinF* expression levels in the hundreds of units; however, nanomolar-level amounts (nM) of opines are detectable for the biosensors developed in this study. Our results indicate that *noxB* and *ooxB* gene expression levels were higher than that of *pinF*. These biosensors may also have enhanced sensitivity because they are unable to degrade the opines, due to disruption in the oxidase genes.

We also quantified the plant opines found in tumor samples using standard curves prepared using the slopes in the lower concentration ranges (0–100 and 0–50 nM for nopaline and octopine, respectively). The response of the biosensors suggested that the amount of nopaline and octopine were 10–20 and 70–74 mg/g dry weight of potato and tomato tumor tissue, respectively. The biosensors were sufficiently sensitive to detect very small amounts of nopaline and octopine indicating that these opine-based biosensors will be useful for diagnosing small opine amounts in plant gall samples.

The grapevine crown gall caused by *A*. *vitis* is still a major problem in nurseries, and pathogenic strains produce opine vitopine^27^. In addition, the infestation of grapevine with root-knot nematodes is spreading all over the world^28^. Differentiation between these two seems more relevant. Since vitopine catabolism is not induced by nopaline or octopine, our biosensors may not applicable to the diagnosis of grapevine crown gall, but if the same strategy is applied to the vitopine catabolism genes, vitopine biosensors could be developed as well.

The worldwide economic damage caused by nematodes is significant, and quarantines between countries are strengthened to prevent nematode infection. However, no diagnostic kits or equipment are currently available for the diagnosis of nematode disease. Our opine-based biosensor can be applied to easily distinguish *Agrobacterium* crown gall disease from nematode disease.

## Methods

### Bacterial strains, plasmids, growth conditions, and chemicals

The bacterial strains and plasmids used in this study are listed in Table 1. *E*. *coli* strains were cultured at 37 °C in Luria-Bertani (LB) medium with or without 1.5% agar. *A*. *tumefaciens* strains were grown at 28 °C in *Agrobacterium* minimal medium with 0.25% mannitol (ABM) as a carbon source. Chemicals and antibiotics were purchased from Sigma-Aldrich and Fisher Scientific (St. Louis, MO, USA). When required, appropriate antibiotics were added to media: for *E*. *coli*, ampicillin (50 μg/mL) and kanamycin (50 μg/mL), and for *A*. *tumefaciens*, kanamycin (100 μg/mL). 5-bromo-4-chloro-indolyl-*β*-d-galactopyranoside (X-gal) was added to 40 μg/mL. Octopine was purchased from Sigma-Aldrich and Fisher Scientific and nopaline was synthesized (Basnet A, Department of Chemistry, Indiana University Bloomington) according to previously reported protocols^29–33^.

**Table 1:** Bacterial strains and plasmids used in this study.

Strain/Plasmid	Characteristics	Reference
***Agrobacterium tumefaciens*** **strains**		
C58	Wild type	
15955	Wild type	
R18	Wild type isolated from raspberry crown gall	This study
*noxB*-*lacZY*	Km^r^, C58 *noxB-lacZY* transcriptional fusion	This study
*ooxB*-*lacZY*	Km^r^, 15955 *ooxB-lacZY* transcriptional fusion	This study
***Escherichia coli*** **strains**		
DH5α	Cloning host	Gibco-BRL
S17-1 *λpir*	Cloning host	^38^
**Plasmids**		
pGEM-T Easy	Amp^r^, Cloning vector	Promega
pVIK112	Km^r^, R6K suicide vector, *lacZY* for transcriptional fusion	^26^
pBY33	Amp^r^, pGEM-T Easy, *A*. *tumefaciens* C58 *noxB* internal fragment	This study
pBY34	Km^r^, pVIK112::*noxB* internal fragment, transcriptional fusion	This study
pBY35	Amp^r^, pGEM-T Easy, *A*. *tumefaciens* 15955 *ooxB* internal fragment	This study
pBY36	Km^r^, pVIK112::*ooxB* internal fragment, transcriptional fusion	This study

Amp^r^: ampicillin resistant, Km^r^: kanamycin resistant.

### General DNA manipulation

DNA manipulation, cloning, restriction enzyme digestion and agarose gel electrophoresis were performed using standard techniques. For most techniques, the procedures described by Sambrook and Russell^34^ were used. DNA sequencing was performed using an ABI3730 sequencer. Restriction enzymes and DNA-depleting enzymes (New England Biolabs, MA, USA and TaKaRa, Otsu, Japan) were used according to the manufacturers’ recommendations. DNA fragments were purified from agarose gel using a QIAEX II gel extraction kit (Qiagen, Hilden, Germany) according to the manufacturer’s instructions. DNA amplification by PCR was performed using a T100 thermal cycler (Bio-Rad, Hercules, CA, USA).

### Construction of *lacZY* transcriptional integration

The plasmid pBY34, carrying the internal fragment of the *noxB* gene was constructed using C58 genomic DNA as the PCR template and NoxBE (5′-GAATTCGCAATTGGATACGGGTTA-3′) and NoxBK (5′-GGTACCGCGATAGTCAGGATGAAT-3′) as primers. The amplified region (318 bp) was purified from agarose gel and ligated into the pGEM-T Easy Vector System (Promega, Mannheim, Germany) to generate pBY33, and the correct sequence was confirmed by sequencing. *EcoR*I-*Kpn*I fragments of pBY33 digested with appropriate restriction enzymes (TaKaRa) were purified after electrophoresis from an agarose gel and inserted into the suicide vector pVIK112^26^, generating pBY34. The resulting construct pBY34 was transferred into *E*. *coli* S17-1 *λpir*, introduced into *A*. *tumefaciens* C58 by conjugation, generating *noxB*-*lacZY* (Fig. 1a). The *lacZY* reporter gene fusion insertion mutants were selected based on the kanamycin-resistance phenotype and confirmed using PCR analysis with primers that annealed upstream of the truncated fragments of *noxB*, NoxPro (5′-TTCGAGACAGCCATTGTT-3′) and LacFuse (5′-GGGGATGTGCTGCAAGGCG-3′).

The plasmid pBY36, carrying the internal fragment of the *ooxB* gene was constructed using 15955 genomic DNA as the PCR template and OoxBE (5′-GAATTCCAGCAAGACGGAGCATTT-3′) and OoxBK (5′-GGTACCGCGTGACAGGATAGAAAA-3′) as primers. The amplified region (345 bp) was purified from agarose gel and ligated into the pGEM-T Easy Vector System (Promega) to generate pBY35, and the correct sequence was confirmed by sequencing. *EcoR*I-*Kpn*I fragments of pBY35 digested with appropriate restriction enzymes (TaKaRa) were purified after electrophoresis from an agarose gel and inserted into the suicide vector pVIK112, generating pBY36. The resulting construct pBY36 was transferred into *E*. *coli* S17-1 *λpir*, introduced into *A*. *tumefaciens* 15955 by conjugation, generating *ooxB*-*lacZY* (Fig. 1b). Reporter insertion mutants were selected based on the kanamycin-resistance phenotype and confirmed using PCR analysis with primers that annealed upstream of the truncated fragments of *ooxB*, OoxBPro (5′-ATGGCAAACACCCTGCTG-3′) and LacFuse.

### Plant tumor induction

Potato tumors were artificially induced by inoculation with *A*. *tumefaciens* C58 and 15955 as previously reported^35^. Briefly, five 1-cm-diameter potato tissue discs were placed on water agar, and each was inoculated with 100 μL of suspension of the *Agrobacterium* strains C58 and 15955. The inoculated discs were incubated at room temperature for 3–4 weeks. To induce tumors in healthy tomato plants, a V-shaped wound (10–60 mm in length) was made in the middle of a young internode using a razor blade. The wound was inoculated with concentrated bacterial suspensions of *A*. *tumefaciens* strains C58 and 15955. The wounds were wrapped with wet gauze, sealed with parafilm, and wet treated for 1 week. The inoculated plants were incubated at room temperature for 5–6 weeks.

### Crude opine extraction from plant tumors

Crude opines were extracted from tumors of potato, tomato and raspberry as previously described^36^ but with modifications. The tumor samples were dried at room temperature for 2 days. The dried tumor samples were ground and dissolved in sterile distilled water (0.01 g tumor/mL). After 1 h, the supernatant was centrifuged and filtered using a 0.45 μm filter (Sartorius, Gottingen, Germany).

### Bacterial culture assay

The responses of opine biosensor strains to synthetic nopaline and octopine and plant gall tissues were analyzed using the imbedding and streaking method. In the first method, tumor tissues or paper discs containing chemical opines were placed onto a biosensor strain imbedded in an ABM agar plate containing X-gal (ABMX-gal). In the second method, a biosensor strain was streaked close to the tumor or chemical samples on an ABMX-gal.

### Thin layer chromatography (TLC) assay

Prior to analytical TLC, samples (1–5 μL) were applied to reverse-phase TLC plates (Merck, Darmstadt, Germany), and chromatograms were developed using chloroform/acetic acid/water (2.8:3.5:0.7, v:v:v). After development, the solvent was evaporated, and the dried plates were overlaid with ABMX-gal agar media containing both biosensor strains (1/100 volume of medium), which were overnight cultured in ABM broth separately, plus 100 μg/mL kanamycin and incubated at 28 °C.

### β-Galactosidase activity assay

A liquid-culture based β-galactosidase activity assay using ortho-nitrophenyl-β-galactoside (ONPG) as a colorimetric substrate was performed as previously described^37^. Mid-log phase biosensor strain cultures were diluted 1:100 with ABM broth to an OD_600_ of 0.01, mixed with different concentrations of nopaline or octopine (1–500 nM) in 2-mL of ABM broth, and incubated at 28 °C to an OD_600_ of ~0.6. Mid-log phase cultures were used for subsequent assays.
